# Arginine vasopressin altered the expression of monocarboxylate transporters in cultured astrocytes isolated from stroke-prone spontaneously hypertensive rats and congenic SHRpch1_18 rats

**DOI:** 10.1186/s12974-017-0949-8

**Published:** 2017-09-02

**Authors:** Kazuo Yamagata, Natsumi Takahashi, Nozomi Akita, Toru Nabika

**Affiliations:** 1 Laboratory of Molecular Health of Food, Department of Food Bioscience and Biotechnology, College of Bioresource Sciences, Nihon University (NUBS), 1866, Kameino, Fujisawa, Kanagawa 252-8510 Japan; 2 Department of Functional Pathology, Shimane University Faculty of Medicine, Matsue, Japan

**Keywords:** Astrocytes, BDNF, SHRSP, MCT4

## Abstract

**Background:**

Astrocytes support a range of brain functions as well as neuronal survival, but their detailed relationship with stroke-related edema is not well understood. We previously demonstrated that the release of lactate from astrocytes isolated from stroke-prone spontaneously hypertensive rats (SHRSP/Izm) was attenuated under stroke conditions. The supply of lactate to neurons is regulated by astrocytic monocarboxylate transporters (MCTs). The purpose of this study was to examine the contributions of arginine vasopressin (AVP) and/or hypoxia and reoxygenation (H/R) to the regulation of MCTs and neurotrophic factor in astrocytes obtained from SHRSP/Izm and congenic SHRpch1_18 rats.

**Methods:**

We compared AVP-induced lactate levels, MCTs, and brain-derived neurotrophic factor (BDNF) in astrocytes isolated from SHRSP/Izm, SHRpch1_18, and Wistar Kyoto rats (WKY/Izm). The expression levels of genes and proteins were determined by PCR and Western blotting (WB).

**Results:**

The production of lactate induced by AVP was increased in astrocytes from all three strains. However, the levels of lactate were lower in SHRSP/Izm and SHRpch1_18 animals compared with the WKY/Izm strain. Gene expression levels of *Slc16a1*, *Slc16a4*, and *Bdnf* were lowered by AVP in SHRSP/Izm and SHRpch1_18 rats compared with WKY/Izm. The increase of MCT4 that was induced by AVP was blocked by the addition of a specific nitric oxide (NO) chelator, 2-(4-carboxyphenyl)-4,4,5,5-tetramethylimidazoline-1-oxyl-3-oxide (CPTIO). Furthermore, AVP increased the expression of iNOS and eNOS proteins in WKY/Izm and SHRSP/Izm rat astrocytes. However, the iNOS expression levels in SHRSP astrocytes differed from those of WKY/Izm astrocytes. The increase of MCT4 protein expression during AVP treatment was blocked by the addition of a specific NF-kB inhibitor, pyrrolidine dithiocarbamate (PDTC). The induction of MCT4 by AVP may be regulated by NO through NF-kB.

**Conclusions:**

These results suggest that the expression of MCTs mediated by AVP may be regulated by NO. The data suggest that AVP attenuated the expression of MCTs in SHRSP/Izm and SHRpch1_18 astrocytes. Reduced expression of MCTs may be associated with decreased lactate production in SHRSP.

## Gene approved symbols

MCT1; *SLC16A1*, MCT4; *SLC16A4*, BDNF; *Bdnf*

## Background

Stroke-prone spontaneously hypertensive rats (SHRSP) develop severe hypertension and die of a stroke [1]. Following the induction of a stroke (ischemic conditions), neuronal cell death [2], and astrocytic edema [3] are observed in SHRSP/Izm animals (where “Izm” indicates the maintaining institution). In SHR brains, the expression of MCTs occurs after occlusion of the middle cerebral artery (MCAO) [4]. The SHRpch1_18 rat was produced by introducing the quantitative trait loci (QTLs) of chromosomes 1 and 18 from SHRSP/Izm into SHR/Izm [5]. SHRpch1_18 rats have salt sensitivity that may enhance stroke onset. In addition, we demonstrated that the release of lactate was significantly lower in astrocytes isolated from SHRSP/Izm rats than Wistar Kyoto rats/Izm (WKY/Izm) under ischemic conditions such as hypoxia and reoxygenation (H/R) [2, 6, 7].

Brain lactate is released from astrocytes under ischemic conditions. It is utilized as the sole energy substrate supporting neuronal functions [8]. A reduced supply of lactate from astrocytes under pathologic conditions, such as ischemia, is associated with neuronal cell death [9]. Following ischemic stimulation, the supply of lactate provided by astrocytes to neurons is regulated by several monocarboxylate transporters (MCTs) [10, 11]. MCTs are transporters of monocarboxylates such as lactate, pyruvate, and ketone bodies [12]. In the brain, three MCT isoforms have been identified: MCT1, MCT2, and MCT4. Within the brain, MCT1 is present in astrocytes, endothelial cells of blood vessels, and ependymocytes [12, 13]. In contrast, MCT2 is expressed by cultured brain neurons [12, 13]. MCT1 and MCT4 are strongly expressed by cortical astrocytes. These results suggest that astrocytes supply lactic acid to neuronal cells [12, 13]. Specifically, MCT1 and MCT4 regulate lactate release by astrocytes, whereas MCT2 regulates lactate uptake by neuronal cells [13]. On the other hand, dysfunction of these astrocytic MCTs may attenuate lactate production and increase neuronal injury [14].

Brain-derived neurotrophic factor (BDNF) regulates neuronal cell growth, neuronal synaptic plasticity, long-term memory, and neuronal cell survival [15]. Furthermore, in rats, BDNF enhances blood pressure in the hypothalamus (PVN) by angiotensin signaling [16]. In rat astrocytes, the anti-epileptic drug valproate enhances the expression of BDNF [17] and inducible nitric oxide synthase (iNOS), and they regulate inflammation and neuronal cell death under pathological conditions [18]. In addition, concentrations of BDNF are reduced in the acute phase of ischemic strokes, and it is associated with the risk of stroke onset [19]. In SHRSP strains, a mutation of the *TrkB* gene, which encodes the receptor for BDNF, has been found [20]. In particular, BDNF plays an important role in promoting neuronal survival [21, 22]. During ischemia, lactate and BDNF production may be regulated by astrocytes [23].

Arginine-vasopressin (AVP) induces production of inflammatory molecules after cerebral edema [24], and it is associated with disruption of the blood-brain barrier (BBB) [25]. In addition, AVP enhances ischemia-evoked edema in the cortex in ischemic strokes [26]. It was further demonstrated that the AVP V1 receptor inhibitor reduced ischemia-induced cerebral edema following stroke [27]. In particular, AVP influences astrocytic function, thereby contributing to the onset of stroke [28]. Under ischemic conditions, AVP might be related to augmented inflammation and serine production, and perhaps stroke in SHRSP/Izm rats [29, 30]. However, there is little understanding regarding AVP and MCT expression. For example, AVP-induced events (such as cerebral edema) are associated with stroke. However, in SHRSP astrocytes, the relationship between lactate control and BDNF expression by AVP is not known. We hypothesized that the reduction of lactate or BDNF expression by AVP might be related to the induction of stroke in the SHRSP/Izm rat strain. The purpose of this study was to examine the contributions of AVP and/or H/R to the regulation of MCTs and neurotrophic factor in astrocytes from SHRSP/Izm and congenic SHRpch1_18 rats. Here, we compared AVP-induced lactate levels, MCTs, and BDNF in astrocytes isolated from SHRSP/Izm and SHRpch1_18 and WKY/Izm rats.

## Methods

### Cell cultures and treatments

Primary dissociated astrocytes were isolated from fetal cerebral WKY/Izm, SHR/Izm, and SHRSP/Izm rats (Japan SLC, Inc., Tokyo, Japan) and the congenic rat strain, SHRpch1_18, as described previously [30, 31]. The SHRpch1_18 strain was produced by introducing the quantitative trait loci (QTL) for stroke sensitivity on chromosomes 1 and 18 of SHRSP/Izm into SHR/Izm [5]. Cultured cells consisted of > 95% astrocytes as determined by glial fibrillary acidic protein (GFAP, Doka Japan, Japan) staining. Astrocytes were plated on 90-mm culture dishes and cultured in Dulbecco’s modified Eagle’s medium (DMEM, Sigma, Japan) containing 10% fetal bovine serum (FBS, Sigma), penicillin (100 U/mL, Life Technologies, Japan), and streptomycin (100 μg/mL, Life Technologies, Sigma-Aldrich) until they reached confluence at 37 °C in a CO_2_ incubator (95% air and 5% CO_2_). The studies described here were approved by the Nihon University animal care and use committee (Approval number; AP15B068).

Astrocytes were seeded on 90-mm, 24-well culture plates (Sumitomo Bakelite Co., LTD, Tokyo, Japan) at an initial density of 40 × 10^4^ cells per cm^2^ and were grown in DMEM containing 10% FBS until confluence was reached. For L-serine measurement, the confluent astrocytes were growth-arrested for 1 day in L-serine-free DMEM (GIBCO BRL, no. 61100) containing 0.2% FBS. Subsequently, the astrocytes’ medium was changed to 100 nM AVP-supplemented (Sigma-Aldrich) or L-serine-free, 0.2% FBS-supplemented DMEM lacking AVP as a control. Hypoxia and reoxygenation stimulation exposed the cells to hypoxic conditions. Namely, the astrocytes were cultured in 1% O_2_, 94% N_2_, and 5% CO_2_ in a CO_2_ incubator (Wakenyaku, Co, Ltd., Japan) for 24 h. Subsequently, the astrocytes were stimulated in air (21% O_2_) and 5% CO_2_ for reoxygenation (30 min or 2 h), as indicated previously [2, 6, 7]. The confluent growth of astrocytes was treated with or without 2-(4-carboxyphenyl)-4,4,5,5-tetramethylimidazoline-1-oxyl-3-oxide (CPTIO, Dojindo Laboratories, Japan, 200 μM) or pyrrolidine dithiocarbamate (PDTC, Sigma-Aldrich) for 60 min before addition of AVP and 20 μM sodium nitroprusside (SNP, Wako Pure Chemical Inc., Japan).

### Measurement of lactate contents in astrocyte-conditioned medium

The lactate content of astrocyte-conditioned medium was measured with an assay kit (Boehringer Mannheim, IN, USA). Protein levels were measured using the Bio-Rad protein assay kit (Bio-Rad, CA, USA), based on the Lowry method, with bovine serum albumin (BSA) as a standard.

### Extraction of total RNA and cDNA synthesis

Total RNA was isolated from cultured astrocytes using TRIzol reagent (Life Technologies Japan Ltd., Tokyo, Japan). DNase I (Life Technologies Japan Ltd.) was added at room temperature for 20 min to remove genomic DNA. DNase I was treated for 15 min at 65 °C to inactivate enzyme activity. Synthesis of the first-strand cDNA was performed using the Superscript III kit (Life Technologies, Japan Ltd.).

### Reverse transcription- polymerase chain reaction (PCR) and quantitative PCR

RT-PCR was carried out to analyze the expression of genes as described in our previous study [30]. After the reaction, gene amplicons were analyzed by 2% agarose electrophoresis (FMC Products, Rockland, ME, USA) and visualized with UV illumination after staining with ethidium bromide. Gene expression levels were evaluated relative to 18S ribosomal RNA (rRNA). Quantitative PCR was performed with the Sequence Detector System (Applied Biosystems, Foster City, CA) [32], as described previously [6]. Quantitative PCR was carried out for MCT1 (*Slc16A1*), MCT4 (*Slc16A4*), and BDNF (*Bdnf*) and to monitor the expression of a housekeeping gene, 18S rRNA (rRNA). Serial dilutions (1:5) of cDNA were used to create a standard curve for the quantitation of gene expression. Primers and TaqMan probes were designed with a primer design software, Primer Express (Applied Biosystems). The forward primer was 5′-TCGTTGGACCCCAGAGGT-3′ for *Slc16a1*, and the reverse primer was 5′-AGGACAGGACAACATTCCACA-3′. The primers for *Slc16a1* amplified a fragment of 67 bp. The sequence of the TaqMan probe was 5′-CAGTGCTGTGGGCTTGGTGACCA-3′ for *Slc16a1*. The forward primer for *Slc16a4* was 5′-CAGGTTTTTGGGATATGCCA-3′. The reverse primer for *Slc16a4* was 5′-TCCAGCCTGCT ATTGGTGG-3′. The primer sequence for *Slc16a4* amplified a fragment of 71 bp. The sequence of the TaqMan probe was 5′-TTTCTTTGCTGGGA TGGCTGTTCTTTC-3′ for *Slc16a4*. The forward primer chosen for *Bdnf* was 5′-CCATAAGGACGCGGACTTG-3′. The reverse primer was 5′-GAGCAGAGGAGGCTCCAAAG-3′. The primer sequence for *Bdnf* amplified a fragment of 73 bp. The sequence of the TaqMan probe was 5′-TCCCGGGTGATGCTCAGCAGTC-3′ for *Bdnf*. The details of quantitative PCR were outlined in our previous report [6] of RT-PCR were confirmed by quantitative PCR.

### Evaluation of protein expression by Western blot

Astrocytes were lysed in RIPA buffer (Thermo Fisher Scientific K.K. Tokyo, Japan, 25 mM Tris-HCl pH 7.6, 150 mM NaCl, 1% NP-40, 1% sodium deoxycholate, and 0.1% SDS) containing a complete protease inhibitor cocktail (Roche Diagnostics). Cell lysates (30 μg protein/line) were assessed by SDS-PAGE and transferred to polyvinylidene difluoride membranes. Membranes were treated with a horseradish peroxidase-conjugated antibody (Life Technologies), and expression levels were examined using an enhanced chemiluminescence system (Life Technologies) with a C-Digit blot scanner (MS Techno Systems Inc. Tokyo, Japan). Antibodies for anti-MCT1 antibody (1: 200, Santa Cruz Biotechnology, USA: sc-50325), anti-MCT4 (1:200, Santa Cruz Biotechnology, USA: sc-50329), anti-iNOS (1:500, Abcam, ab 3523), anti-eNOS (1:1000, Cell Signaling Technology, The Netherlands: #32027), and β-actin (1:1000, Cell Signaling Technology: #4967) were used.

### Statistical analysis

Data are presented as means ± SE. The significance of differences was determined using Fisher’s protected least significant difference (PLSD) method following an analysis of variance (ANOVA).

## Results

### Effect of AVP on the production of lactate by astrocytes isolated from WKY/Izm, SHRSP/Izm, and SHRpch1_18h rats

**Fig. 1:**
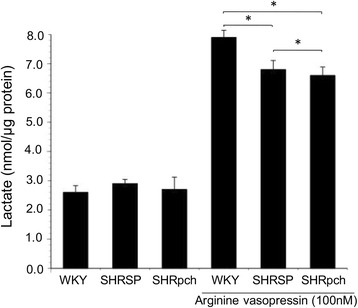
Effect of AVP on lactate production by astrocytes isolated from WKY/Izm, SHRSP/Izm, and SHRpch1_18 rats*.* Confluent astrocytes isolated from WKY/Izm, SHRSP/Izm, and SHRpch1_18 rats were exposed to 0 or 100 nM AVP for 24 h. WKY, WKY/Izm; SHRSP, SHRSP/Izm; SHRpch; SHRpch1_18. Columns show means ± SE (*n* = 4). *P < 0.05

Astrocytes provide lactate to neuronal cells for use as an energy substrate. Astrocytes isolated from WKY/Izm, SHRSP/Izm, and SHRpch1_18 strains were treated with AVP, and we determined the individual levels of lactate. In WKY/Izm, SHRSP/Izm, and SHRpch1_18 astrocytes, addition of 100 nM AVP enhanced the level of lactate production during the 24 h treatment (Fig. 1). However, the level of production of lactate was significantly (p < 0.05) lower in SHRSP/Izm and SHRpch1_18 strains than in WKY/Izm astrocytes at 100 nM AVP. Specifically, after 24 h, AVP-enhanced lactate production increased about 3.03-fold (WKY/Izm), 2.34-fold (SHRSP/Izm), and 2.44-fold (SHRpch1_18) above the control level in astrocytes isolated from WKY/Izm, SHRSP/Izm, and SHRpch1_18 rats, respectively. Effect of AVP on gene expression levels of Slc16a1, Slc16a4, and Bdnf in astrocytes isolated from WKY/Izm, SHRSP/Izm, and SHRpch1_18 rats.

### Effect of AVP on Slc16a1, Slc16a4, and Bdnf gene expression in astrocytes from WKY/Izm, SHRSP/Izm, and SHRpch1_18 rats

**Fig. 2:**
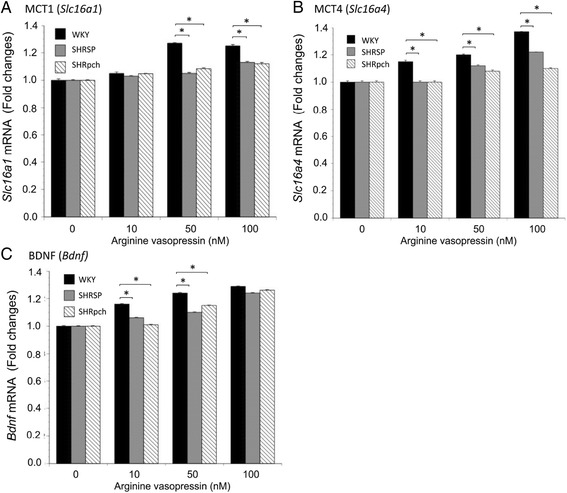
Effect of AVP on *Slc16a1*, *Slc16a4*, and *Bdnf* gene expression in astrocytes from WKY/Izm, SHRSP/Izm, and SHRpch1_18 rats. Confluent astrocytes isolated from SHRSP/Izm, SHRpch1_18 rats, and WKY/Izm were exposed to 10, 50, or 100 nM AVP for 8 h, after which total cellular RNA was used for RT-PCR analysis. Comparison of **a** *Slc16a1*, **b** *Slc16a4*, and **c** *Bdnf* gene expression in astrocytes from all assayed rat strains. Gene expression levels were normalized to 18S ribosomal RNA. WKY WKY/Izm, SHRSP SHRSP/Izm, SHRpch SHRpch1_18. Columns show means ± SE (*n* = 4). *P < 0.05

**Fig. 3:**
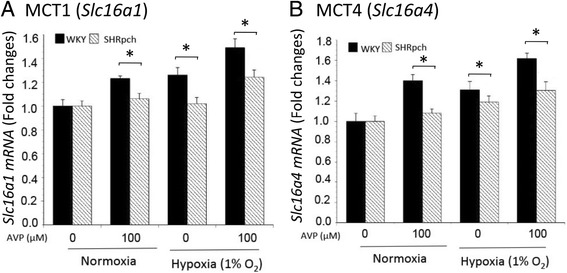
Effect of AVP during normoxia and hypoxia on *Slc16a1* and *Slc16a4* gene expression in astrocytes isolated from WKY/Izm and SHRpch1_18 rats. Confluent astrocytes isolated from WKY/Izm and SHRpch1_18 rats were exposed to normoxic (21% O_2_) or hypoxic (1% O_2_) conditions for 24 h with or without 100 nM AVP. Total cellular RNA was isolated from the cultured astrocytes for RT-PCR, using primers for *Slc16a1* or *Slc16a4*. **a** *Slc16a1* and **b** *Slc16a4* gene expression from astrocytes from all assayed rat strains. Columns show means ± SE (*n* = 4). Normoxia 21% O_2,_ hypoxia 1% O_2_. AVP arginine vasopressin. *P < 0.05

The expression of a lactate transport gene (*Slc16a1*) was compared in astrocytes isolated from three strains (WKY/Izm, SHRSP/Izm, and SHRpch1_18h) following 8 h treatment with AVP (10, 50, and 100 μM). In astrocyte of three strains, AVP increased gene expression of *Slc16a1* (Fig. 2a). However, the gene expression level of *Slc16a1* in astrocytes was significantly lower (p < 0.05) in cells from SHRSP/Izm and SHRpch1_18 rats than in those from WKY/Izm rats at 10, 50, and 100 nM AVP (p < 0.001). In 100 nM AVP, enhanced gene expression was about 1.25-fold, 1.13-fold, and 1.12-fold the control level in astrocytes isolated from WKY/Izm, SHRSP/Izm, and SHRpch1_18 rats, respectively. Similarly, the expression of the *Slc16a4* gene was compared in astrocytes isolated from three strains following an 8 h treatment with AVP (10, 50, and 100 nM) (Fig. 2). In all astrocytes, AVP increased gene expression of *Slc16a4*. However, gene expression of *Slc16a4* was significantly lower in SHRSP/Izm (1.0-, 1.12-, and 1.22-fold) and SHRpch1_18 (1.0-, 1.08-, and 1.1-fold) than in WKY/Izm (1.15-, 1.2-, and 1.37-fold) rat astrocytes at 10, 50, and 100 nM of AVP (p < 0.05). Furthermore, the expression levels of genes for neurotrophic factor *Bdnf* were compared in astrocytes isolated from the three strains after 8 h exposure to AVP (10, 50, and 100 μM) (Fig. 3). In astrocytes isolated from the three strains, gene expression levels of *Bdnf* were significantly increased by addition of AVP in a dose-dependent manner. In WKY/Izm astrocytes, increased levels were 1.16-, 1.24-, and 1.28-fold higher in 10, 50, and 100 nM AVP compared with basal levels, respectively (Fig. 2c). However, the gene expression levels of *Bdnf* were significantly lower in cells from the SHRSP/Izm (1.06-, 1.10-, and 1.24-fold) and SHRpch1_18 (1.01-, 1.15-, and 1.26-fold) strains than those from WKY/Izm (1.16-, 1.24-, and 1.28-fold) rat astrocytes at 10, 50, and 100 nM of AVP (p < 0.05). These results were confirmed by real-time PCR. Thus, the gene expression levels of *Slc16a1* and *Slc16a4* were significantly lower (p < 0.05) in cells from the SHRSP/Izm and SHRpch1_18 strains than those from WKY/Izm rat astrocytes at 50 and 100 nM AVP.

### Effect of AVP during normoxia and hypoxia on Slc16a1 and Slc16a4 gene expression in astrocytes isolated from WKY/Izm and SHRpch1_18 rats

AVP and hypoxia induce astrocytic edema [33]. Therefore, cultured astrocytes isolated from WKY/Izm and SHRpch1_18 rats were exposed to 21% O_2_ (normoxic conditions) or 1% O_2_ (hypoxic conditions) for 24 h with or without 100 nM AVP. After 24 h normoxic conditions, *Slc16a1* gene expression levels in WKY/Izm astrocytes were significantly (*p* < 0.05) increased by AVP treatment compared to control cells, but this was not observed in SHRpch1_18 cells (Fig. 3a). Following 24 h hypoxic conditions, *Slc16a1* gene expression levels in WKY/Izm and SHRpch1_18 astrocytes were significantly (*p* < 0.05) increased by AVP treatment compared to control cells. However, expression of *Slc16a1* was significantly (p < 0.05) lower in SHRpch1_18 rat astrocytes than in WKY/Izm rat astrocytes in 100 nM AVP (Fig. 3a). In addition, the level of gene expression of *Slc16a1* was significantly (p < 0.05) lower in SHRpch1_18 astrocytes than WKY/Izm astrocytes under hypoxic conditions. Similarly, gene expression of *Slc16a4* was examined. As shown in Fig. 3b, after 24 h of normoxia, *Slc16a4* gene expression levels in WKY/Izm rat astrocytes were significantly (*p* < 0.05) increased by AVP treatment compared to control cells, but this did not occur in SHRpch1_18 cells. Under hypoxic conditions, *Slc16a4* gene expression in astrocytes isolated from WKY/Izm and SHRpch1_18 rats was significantly (*p* < 0.05) increased by AVP treatment compared to control cells. On the other hand, gene expression of *Slc16a4* was significantly lower (p < 0.05) in SHRpch1_18 than in WKY/Izm rat cells at 100 nM AVP in normoxic and hypoxic conditions.

### Effect of AVP during hypoxia and reoxygenation (H/R) on Slc16a1 and Slc16a4 gene expression in astrocytes isolated from WKY/Izm and SHRpch1_18 rats

**Fig. 4:**
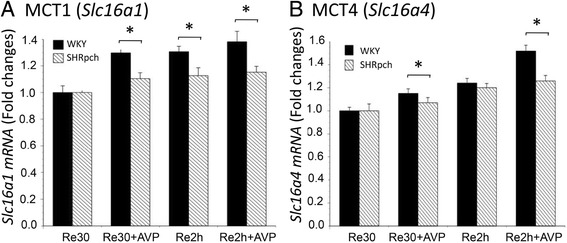
Effect of AVP during hypoxia and reoxygenation (H/R) on *Slc16a1* and *Slc16a4* gene expression in astrocytes isolated from WKY/Izm and SHRpch1_18 rats. Confluent astrocytes isolated from WKY/Izm and SHRpch1_18 rats were exposed to hypoxic (1% O_2_) conditions for 24 h with or without 100 nM AVP. After hypoxic culture, they were maintained under normoxic conditions for 30 min or for 2 h with or without 100 nM AVP. Total cellular RNA was isolated from the cultured astrocytes for RT-PCR, using primers for *Slc16a1* or *Slc16a4*. **a**. *Slc16a1* and **b**. *Slc16a4* gene expression from astrocytes from all assayed rat strains. Columns show means ± SE (*n* = 4). Re30, after hypoxic culture (1% O_2_), cells were maintained in normoxic conditions (21% O_2_) for 30 min; Re2h, after hypoxic culture (1% O_2_), cells were maintained in normoxic conditions (21% O_2_) for 2 h. AVP 100 nM arginine vasopressin. *P < 0.05

In additional experiments, astrocytes isolated from WKY/Izm and SHRpch1_18 rat strains were incubated in 1% O_2_ for 24 h and then transferred to 21% O_2_ for 30 min or 2 h; 100 nM AVP was added before both hypoxia and reoxygenation. In 30 min of reoxygenation following 24 h of hypoxia, *Slc16a1* gene expression in astrocytes isolated from WKY/Izm and SHRpch1_18 rats was significantly (*p* < 0.05) increased by AVP treatment compared to control cells (without AVP) (Fig. 4a). However, in 100 nM AVP, expression of the *Slc16a1* gene was significantly (p < 0.05) lower in WKY/Izm cells than in those from the SHRpch1_18 strain. Furthermore, after 2 h of reoxygenation following 24 h of hypoxia, *Slc16a1* expression levels in WKY/Izm and SHRpch1_18 astrocytes were significantly (*p* < 0.05) increased by AVP treatment compared to control cells. Expression of *Slc16a1* was significantly lower (p < 0.05) in SHRpch1_18 than in WKY/Izm rat astrocytes in 100 nM AVP (Fig. 4a). Following the addition of AVP, gene expression of *Slc16a1* did not change compared with cells lacking AVP.

Similarly, expression of *Slc16a4* was examined with hypoxia and H/R conditions (Fig. 4b). Following 24 h of hypoxia and 30 min of reoxygenation, *Slc16a4* expression levels in astrocytes isolated from WKY/Izm and SHRpch1_18 rats were significantly (*p* < 0.05) increased by AVP treatment compared to control cells (without AVP) (Fig. 4a). However, expression of *Slc16a4* was significantly lower (p < 0.05) in SHRpch1_18 than in WKY/Izm rat cells in 100 nM AVP (Fig. 4b). Following 24 h of hypoxia and 2 h of reoxygenation, *Slc16a4* gene expression levels in astrocytes isolated from WKY/Izm and SHRpch1_18 rats were significantly (*p* < 0.05) increased by AVP treatment compared to control cells. Gene expression of *Slc16a4* was significantly lower (p < 0.05) in cells from SHRpch1_18 rats than those from WKY/Izm rats at 100 nM AVP. These results were confirmed in real-time PCR. Thus, AVP attenuated the expression of MCTs in SHRpch1_18 astrocytes during hypoxia and H/R conditions.

### Effect of AVP on the expression of MCT4 protein in astrocytes isolated from WKY/Izm, SHRSP/Izm, and SHRpch1_18 rats

**Fig. 5:**
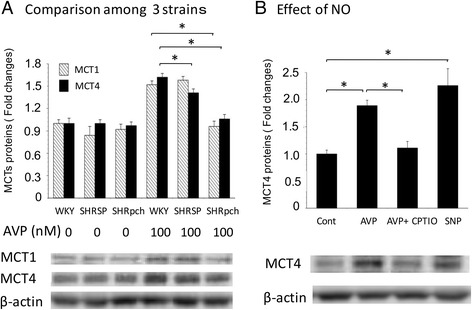
Effect of AVP on expression of MCT4 protein in astrocytes isolated from WKY/Izm, SHRSP/Izm, and SHRpch1_18 rats. Astrocytes isolated from WKY/Izm, SHRSP/Izm, and SHRpch1_18 rats were exposed to 0 or 100 nM AVP for 24 h, after which MCT4 protein was assessed by WB analysis (**a**). In addition, astrocytes isolated from WKY/Izm were treated with or without AVP (100 nM), AVP plus CPTIO and SNP for 24 h (**b**). Astrocytes were exposed to CPTIO (200 μM) for 60 min before addition of AVP and 20 μM SNP. Total protein (30 μg) was analyzed by Western blot with an anti-MCT1 antibody or an anti-MCT4 antibody. AVP arginine vasopressin, WKY WKY/Izm, SHRSP SHRSP/Izm, SHRpch SHRpch1_18, AVP arginine vasopressin, SNP sodium nitroprusside. Columns show means ± SE (*n* = 4). *P < 0.05

Astrocytes were isolated from WKY/Izm, SHRSP/Izm, and SHRpch1_18 rats, and we compared the expression of MCT1 and MCT4 proteins after culture in AVP. Specifically, astrocytes were treated with 100 nM AVP for 24 h, and then MCT1 and MCT4 protein levels were examined by WB. As shown in Fig. 5a, expression of MCT1 and MCT4 was increased by AVP treatment of astrocytes isolated from the three rat strains. In 100 nM AVP, astrocytes’ expression of MCT1 protein was slightly lower in cells from SHRSP/Izm and SHRpch1_18 rats than in those from the WKY/Izm strain. Similarly, expression of MCT4 protein was slightly lower in astrocytes from the SHRSP/Izm and SHRpch1_18 strains than in those from WKY/Izm astrocytes.

We next determined whether AVP influenced the effects of MCT4 expression by pre-treating WKY/Izm astrocytes with or without an NO inhibitor. CPTIO is a chelator of NO that blocks the effect of NO [34]. In addition, SNP is a donor of NO that enhances levels of NO [35]. Thus, we investigated whether pre-treating WKY/Izm astrocytes with CPTIO or SNP affected AVP-induced expression of MCT4 protein. As shown in Fig. 5b, MCT4 protein expression in astrocytes was upregulated by 100 nM AVP treatment, and this increase could be inhibited by the addition of CPTIO. Furthermore, SNP treatment increased the expression of MCT4 protein. These results suggest that the expression of MCT4 is related to NO production.

### Effect of AVP on the expression of iNOS and eNOS proteins in astrocytes isolated from WKY/Izm and SHRSP/Izm

**Fig. 6:**
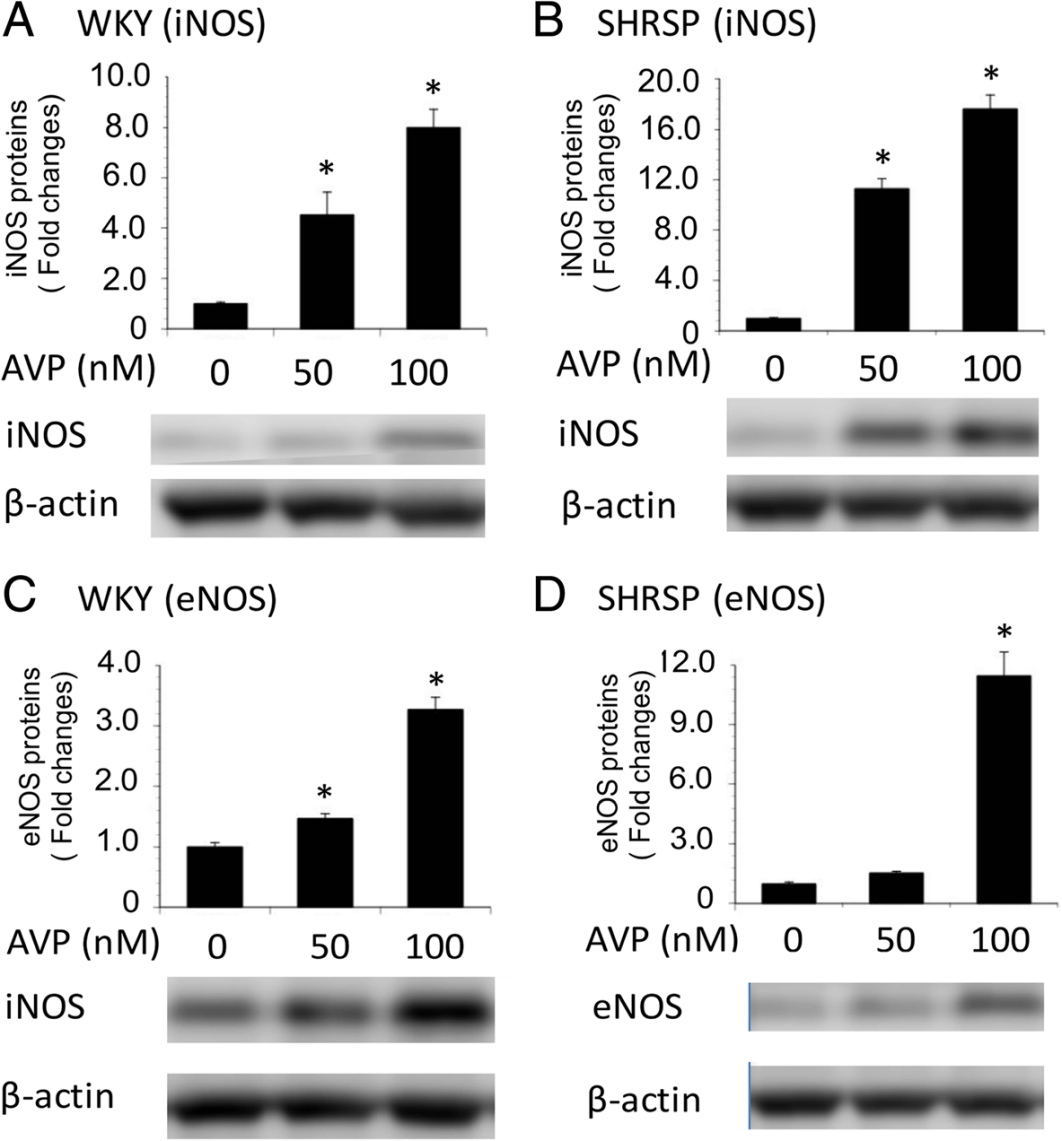
Effect of AVP on expression of iNOS and eNOS proteins of astrocytes isolated from WKY/Izm and SHRSP/Izm rats. Astrocytes isolated from WKY/Izm and SHRSP/Izm rats were exposed to 0 or 100 nM AVP for 24 h, after which iNOS (**a**. WKY/Izm, **b**. SHRSP/Izm) and eNOS (**c**. WKY/Izm, **d**. SHRSP/Izm) protein was assessed by WB analysis. Total protein (30 μg) was analyzed by Western blot with an anti-iNOS or an anti-eNOS antibody. AVP arginine vasopressin, WKY WKY/Izm, SHRSP SHRSP/Izm. Columns show means ± SE (*n* = 4). *P < 0.05

We examined how AVP affected iNOS and eNOS protein expression in WKY/Izm and SHRSP/Izm astrocytes (Fig. 6). AVP significantly increased (p < 0.05) the expression of iNOS protein in WKY/Izm astrocytes (50 nM, 4.0-fold; 100 nM, 7.3-fold) and SHRSP astrocytes (50 nM, 10.6-fold; 100 nM, 16.3-fold) (Fig. 6). In addition, the iNOS expression levels in SHRSP/Izm astrocytes were elevated compared with WKY/Izm at 50 and 100 nM AVP. In WKY astrocytes, eNOS expression with AVP was significantly increased (p < 0.05) in a concentration-dependent manner (50 nM, 1.2-fold; 100 nM, 3.3-fold). However, eNOS expression in SHRSP/Izm was only slightly increased at 50 nM but significantly increased at 100 nM (p < 0.05) (Fig. 6d). The levels of eNOS expression in SHRSP/Izm were lower than in WKY/Izm.

### Effects of PDTC on AVP-induced MCT4 and iNOS protein expression in astrocytes isolated from SHRSP/Izm rats

**Fig. 7:**
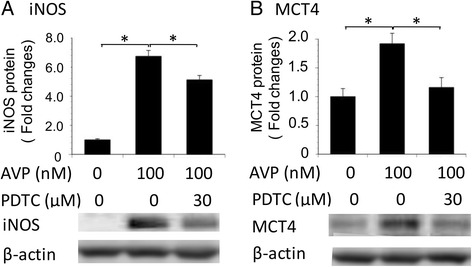
Effects of PDTC on AVP-induced iNOS and MCT4 protein expression in astrocytes isolated from SHRSP/Izm rats. Astrocytes isolated from SHRSP/Izm rats were exposed to 0 or 100 nM AVP for 24 h, after which iNOS (**a**) and MCT4 (**b**) proteins were assessed by WB analysis. In addition, astrocytes isolated from SHRSP/Izm rats were treated with or without AVP (100 nM) or AVP plus PDCT. Astrocytes were exposed to PDCT (30 μM) for 60 min before addition of AVP. Total protein (30 μg) was analyzed by Western blot with an anti-iNOS antibody or an anti-MCT4 antibody. AVP arginine vasopressin. Columns show means ± SE (*n* = 4). *P < 0.05

We investigated how AVP influenced the effects of MCT4 expression by pre-treating SHRSP/Izm astrocytes with or without an NF-kB inhibitor, PDTC [36]. As shown in Fig. 7, AVP increased the expression of iNOS protein in SHRSP astrocytes at 100 nM. The 60 min pretreatment with PDCT-inhibited expression of iNOS protein by AVP (0.79-fold). Also, the pretreatment with PDCT inhibited expression of MCT4 protein by AVP (0.60-fold).

## Discussion

Brain ischemia-induced neuronal cell death is due to a shortage of oxygen and glucose [9, 37]. On the other hand, in the brain, lactate production increases with ischemia [38] and provides neuroprotection [9]. Lactate is produced by astrocytes via glycolysis and glycogenolysis and supports neuronal cells [39]. Regulation of lactate production and transport to neuronal cells by MCTs might reduce neuronal cell death after cerebral ischemia [40]. On the other hand, AVP affects astrocytic functions and regulates the onset of ischemic stroke [28]. For example, AVP induces ischemia-evoked brain edema in the setting of ischemic stroke [26]. Moreover, AVP induces production of inflammatory molecules after cerebral edema [24], and it subsequently enhances disruption of the BBB [25]. To better understand these findings, we compared AVP and/or H/R contributions to lactate production, expression of *Slc16a1*, *Slc16a4*, and *Bdnf* in cultured astrocytes isolated from SHRSP/Izm, SHRpch1_18, and WKY/Izm rats.

A previous report demonstrated that following MCAO in SHR strain rats, the decrease in MCT expression coincided with neuronal cell death in the infarcted area [4]. However, the roles played by these MCTs in strokes in SHRSP strains are not clear. Therefore, in the present study, we assessed the AVP-induced gene expression of Slc16a1 and Slc16a4 in three strains of astrocytes. Expression of *Slc16a1* and *Slc16a4* in SHRSP/Izm and SHRpch1_18 was attenuated by AVP compared with WKY/Izm. These data indicate that the regulation of MCTs could be important for the supply of energy to neurons as well as the regulation of neurodegenerative diseases such as ischemic stroke. MCT1 and MCT4 enhance lactate release from astrocytes and regulate lactate uptake by neuronal cells [13]. Our results suggest that the reduced expression of *Slc16a4* by AVP regulates the supply of lactate to neurons in SHRSP/Izm and SHRpch1_18 rats.

Generally, ischemic conditions induce neurotoxicity and neuronal damage [41]. Importantly, lactate can assist the metabolic activity of hippocampal neuronal cells under hypoxic conditions [42]. Enhanced expression of MCT4 contributed to the resistance to hypoxic injury in astrocyte-neuron co-cultures [11]. In SHRSP strains, the reduced expression of MCT mediated by AVP may be associated with decreased lactate under ischemic conditions. For example, hypoxic and H/R conditions induce neuronal cell death in WKY/Izm and SHRSP/Izm rats [7]. The percentage of neuronal cells that undergo apoptosis during hypoxia-reperfusion is notably higher in SHRSP/Izm rats than in WKY/Izm rats [7]. A previous study analyzed the generation of hydroxyl radicals during hypoxia and reoxygenation in SHRSP rats [43]. In those animals, the generation of hydroxyl radicals was greater than that seen in the WKY strain. Moreover, there was greater oxidative stress, to which neurons are susceptible [2]. Furthermore, under ischemic conditions, AVP might enhance inflammation and attenuate serine production as well as stroke in SHRSP/Izm rats [29, 30]. Therefore, AVP-modulated expression of MCTs in WKY/Izm and SHRpch1_18 astrocytes was examined during hypoxia. In this study, hypoxia stimulation and AVP increased the expression of Slc16a1 and Slc16a4 in astrocytes. These results suggest that ischemic conditions enhanced lactate production that subsequently led to important responses associated with prevention of neuronal cell death. On the other hand, in the presence of AVP, both genes were expressed at significantly lower levels in SHRpch1_18 rat astrocytes than in those from WKY/Izm. These results suggest that ischemic conditions plus AVP addition exacerbated the astrocytic lactic acid supply and subsequently energy deficiency in neuronal cells.

In our study, the expression of *Slc16a1* and *Slc16a4* was examined under hypoxic and H/R conditions. After 30 min of hypoxia and 2 h of reoxygenation, the levels of *Slc16a1* and *Slc16a4* expression in WKY/Izm and SHRpch1_18 astrocytes were increased with and without treatment with AVP. However, the levels of *Slc16a1* and *Slc16a4* expression in AVP were lower in SHRpch1_18 rats than in WKY/Izm rats. On the other hand, it was reported that after the first few minutes of reoxygenation following brain ischemia, ROS (such as free radicals) induce cell damage [44, 45]. In other words, brain ischemia enhances HIF-1α and quickly produces large amounts of ROS, and the subsequent reactions cause cell and brain damage. In particular, in SHRSP/Izm rats, hydroxyl radicals are produced under H/R conditions [43]. In SHRSP/Izm and WKY/Izm rats, the generation of hydroxyl radicals in hippocampal neuronal cells peaked 20 min after the start of H/R. In addition, there was a significantly greater production of hydroxyl radicals in SHRSP/Izm compared with WKY/Izm cells. Our results and these reports appear to suggest sensitive differences for induced ROS production during H/R in SHRSP strains rats.

NO enhances glycolysis in astrocytes [46, 47]. Thus, the presence of NO may generate larger amounts of lactate from astrocytes. In the present study, we examined the contribution of NO to the expression of MCT4 induced by AVP in WKY/Izm cells. Namely, we examined whether AVP increased MCT4 protein expression after pre-treating astrocytes with CPTIO or SNP. In the presence of AVP, MCT4 protein expression in astrocytes was upregulated, and this increase could be inhibited by the addition of CPTIO. On the other hand, treatment with the NO donor, SNP increased the expression of MCT4 protein. One report demonstrated that NO induced the expression of MCT4 in cultured astrocytes [34]. Therefore, expression of MCT4 may be regulated by NO. We investigated whether AVP induced iNOS and eNOS protein expression in WKY/Izm and SHRSP/Izm rat astrocytes. We showed that AVP increased the expression of iNOS and eNOS proteins (Fig. 6). However, the level of expression of eNOS in SHRSP/Izm cells was lower than that in WKY/Izm. Furthermore, induction of MCT4 and iNOS by AVP was blocked by the NF-kB inhibitor PDCT (Fig. 7). Therefore, induction of MCT4 by AVP may be regulated by NO through NFkB. On the other hand, under hypoxic conditions, the increase of lactate production and MCTs may be induced through a transcription factor, hypoxia-inducible factor-1α (HIF-1α) [48]. NOSs may be associated with expression of MCT4 induced in hypoxia [34, 35]. Moreover, AVP, NF-kB, and HIF may be associated with these events. In SHRSP brains, NO production was high, which might explain the susceptibility to neuronal cell injury [49]. However, we previously demonstrated that gene expression of isoforms of 6-phosphofructo-2-kinase (PFK2), a master regulator of glycolysis, was reduced by SNP in SHRSP astrocytes [35]. Specifically, the SNP-induced gene expression of *PFK2.4* was more attenuated in astrocytes from SHRSP rats than in those from WKY. Induction of PFK2.4 by SNP attenuates the glycolytic system and lactate production may decrease. Therefore, in SHRSP cells, expression of MCT by NO may be less than in WKY. These features may be associated with changes in lactic acid, which decreased in SHRSP, although the exact mechanisms responsible for these alterations remain unclear.

BDNF decreases cortical neuronal cell death during post-MCAO ischemia in adult rats [50]. Similarly, BDNF plays a central role in neuronal recovery after cerebral ischemia [51]. Furthermore, 4 weeks after permanent occlusion of bilateral common carotid arteries (CCA), expression of BDNF was lower in SHRSP rats compared to WKY rats [52]. Therefore, we compared the effects of AVP on *Bdnf* gene expression in SHRSP/Izm, SHRpch1_18, and WKY/Izm astrocytes. Here, we showed that *Bdnf* expression in SHRSP/Izm and congenic SHRpch1_18 astrocytes was reduced compared with WKY/Izm rats. *Bdnf* expression in the presence of AVP may be lower in astrocytes from SHRSP/Izm and SHRpch1_18 rats than in astrocytes isolated from WKY/Izm rats. BDNF contributes to several functions such as neuronal cell growth, neuronal synaptic plasticity, long-term memory, and neuronal cell survival [15]. Recently, it was found that BDNF regulates blood pressure in the hypothalamus (PVN) with angiotensin signaling in rats [16]. We reason that the reduced expression of the *Bdnf* gene during AVP treatment may be associated with the attenuated nutritional support in the SHRSP/Izm strain, although the exact mechanisms responsible for these alterations remain unclear. However, these features of SHRSP/Izm and SHRpch1_18 rats may be important contributors to the attenuated neurotrophic supply in astrocytes.

In summary, we demonstrated that cultured astrocytes isolated from SHRSP/Izm and SHRpch1_18 rats differed from those obtained from the WKY/Izm strain in regard to their responsiveness to AVP or H/R conditions. Namely, we found that AVP attenuated lactate production in SHRSP/Izm and SHRpch1_18 astrocytes compared with WKY/Izm. Furthermore, we showed that the expression of MCT1 and MCT4 in astrocytes following AVP treatment or H/R stimulation was decreased. Induction of MCT4 by AVP and hypoxia may be regulated by NO through transcription factor NF-kB activation and HIFα [48]. Previously, we showed that SHRSP rat astrocyte gene expression for LDH and MCT was lower than that in WKY rats [6]. The in vivo study showed that expression of MCT increased in the brain following an ischemic insult [52]. These data might explain the alteration of MCT expression and low lactate production by SHRs strain rat cells in vivo. Thus, in SHRSP/Izm and SHRpch1_18 astrocytes subjected to AVP or H/R stimulation, the specific characteristics of these cells might explain the energy and nutritional deficiencies in neuronal cells.

## Conclusions

The expression of MCTs mediated by AVP may be regulated by NO. AVP attenuated the expression of MCTs in SHRSP/Izm and SHRpch1_18 astrocytes. Attenuated expression of MCTs may be related with decreased lactate production in SHRSP. Reduced production of lactate may be associated with decreased neuronal energy supply in SHRSP/Izm rats. Furthermore, it may play an important role in post-ischemic neuronal recovery [48].

## Abbreviations

AVP: Arginine vasopressin
BDNF: Brain-derived neurotrophic factor
H/R: Hypoxia and reoxygenation
MCT: Monocarboxylate transporter
SHRSP: Stroke-prone spontaneously hypertensive rats
WKY: Wistar Kyoto rats
